# Fecal microbiota colonization dynamics in dairy heifers associated with early-life rumen microbiota modulation and gut health

**DOI:** 10.3389/fmicb.2024.1353874

**Published:** 2024-03-05

**Authors:** Hanna Huuki, Johanna Vilkki, Aila Vanhatalo, Ilma Tapio

**Affiliations:** ^1^Department of Agricultural Sciences, University of Helsinki, Helsinki, Finland; ^2^Production Systems, Genomics and Breeding, Natural Resources Institute Finland (Luke), Jokioinen, Finland

**Keywords:** fecal bacteria, archaea, anaerobic fungi, microbiome modulation, microbiome establishment, heifer, calf, colostrum

## Abstract

Early-life modulation of rumen microbiota holds promise for enhancing calf growth, health, and long-term production in ruminants. However, limited attention has been given to the impact of rumen microbiota modulation on the establishment of hindgut microbiota. In this study, fecal microbiota development was examined in identical twin calves for 12 months. The treatment group (T-group) received adult cow fresh rumen liquid inoculum during the pre-weaning period, while the control group did not (C-group). The effects of inoculum were assessed on calf gut health and as microbial seeding route into the hindgut. The early rumen modulation had no effect on age-related fecal microbiota development. The fecal bacterial community evolved gradually following dietary changes and categorized into pre-weaning and post-weaning communities. Bacterial richness increased with age and stabilized at month 9, while between-sample variation reduced in post-weaning samples. Archaeal load in fecal samples increased after month 4, while archaeal richness increased and stabilized in both groups by month 9. Between-sample similarity was higher during the pre-weaning period, with increased dissimilarity from month 4 onward. Anaerobic fungi were detected in feces at month 4, with richness peaking at month 7. Before month 6, fungal community composition distinctly differed from mature communities. When colostrum, calf rumen, and donor inoculum were evaluated as seeding sources for hindgut colonization, the calf’s own rumen was identified as the primary seeding source for fecal bacteria and fungi. Colostrum was a source for several bacteria detected in feces, but these were of temporary importance until weaning. The donor inoculum had limited impact on gut health as diarrhea rates were similar between the T-group and C-group. In conclusion, early-life microbiota modulation shows potential in ruminant development. However, a more targeted approach with bacteria adapted to the hindgut environment may be necessary to modulate hindgut effectively. This research contributes to our understanding of the complex relationship between gut microbiota and calf health and growth.

## Introduction

The gastrointestinal (GI) tract in young ruminants undergoes both anatomical growth and functional maturation (Baldwin et al., 2004; Guilloteau et al., 2009), which is crucial for nutrient absorption and the development of immune tolerance to symbiotic microbiota (Amin and Seifert, 2021). Upon birth, the first colostrum meal bypasses rumen and is directed toward abomasum, where it helps to create an acidic environment and triggers colostrum digestion and absorption in the small intestine (Lopez and Heinrichs, 2022). Within hours of birth, the gut of a newborn calf allows the passage of colostrum-derived immunoglobulin G, providing short-term immunity. Weaning represents a pivotal stage, transitioning the calf from milk-based digestion in the small intestine into solid feed intake and ruminal fermentation, which will also have an impact on the microbial community (Meale et al., 2016). As solid feed consumption increases, rumen capacity expands significantly, providing an enhanced surface area for the absorption of ruminal fermentation products, particularly VFAs, to meet the calf’s growth demands. Song et al. (2018) hypothesized that hindgut microbial fermentation plays an important role in providing energy to pre-weaned calves prior to the complete development of the rumen by demonstrating significant association between volatile fatty acids (VFAs) and the colonization of mucosa-attached health-related bacterial genera and carbohydrate-utilizing bacterial genera.

Functional development of the rumen is driven by solid feed consumption, while intensive microbial colonization of the GI tract starts at birth. Maternal transfer of microbes occurs during birth through contact with the dam’s vaginal and fecal microbiota and the colostrum, skin, or saliva (Yeoman et al., 2018). These initial microbial populations lay the foundation for the calf’s gut microbiota. Interaction with adult animals further contributes to microbial diversity, as the calf encounters a broader array of microorganisms through close contact and shared living spaces (Lowe et al., 1987; Bird et al., 2010). Further development of GI microbial ecosystem in young ruminants is influenced by diet and use of medication and hygiene practices in housing systems among others. Understanding complex interplay of these factors is crucial for optimizing gut health and overall well-being of young ruminants.

Although the rumen microbial composition and function have been in the center of research (Rey et al., 2014; Furman et al., 2020), the large intestine microbiome may have unexplored potential to improve the animal welfare and production performance (O’Hara et al., 2020). Hindgut microbiota has an important role in immune defense (Hooper et al., 2012), nitrogen metabolism and cycling (Hoover, 1978) or can produce a variety of compounds, e.g., vitamins (Jiang et al., 2022) and bile acid metabolites (Lin et al., 2023), which may have an impact on the host nutrient absorption, metabolism, and health. While ruminants acquire most of their energy from the ruminal fermentation, hindgut fermentation accounts for 5–10% of the total metabolizable energy (Gressley et al., 2011) with some studies suggesting association of fecal microbiota with feed efficiency (Welch et al., 2021). Compared with the rumen, much less is known about the colonization of the large intestine and the potential to modulate its microbial ecosystem through early life interventions. However, some studies have demonstrated successful impact of hindgut microbiota modulation. Yu et al. (2020) orally inoculated lambs with rumen fluid from an adult sheep and showed that inoculation during weaning had stronger effect on the colonic microbiota than the inoculation before weaning. Another study showed mild effects of fecal microbiota transplant from adult cows on microbiota of young calves and ability to alleviate weaning stress through pro-inflammatory response and increasing antioxidant potential (Rosa et al., 2021). Similarly, an oral dose of fecal material obtained from healthy calves was capable of ameliorating diarrhea in sick calves of similar age with alterations in their gut microbiota (Kim et al., 2021). In human studies, the transfer of beneficial microbiota though fecal microbiota transplantation has demonstrated high potential to ameliorate gut dysbiosis associated with *Clostridium difficile* infection, suggesting that colonic microbial modulation leading to health benefits for humans and animals is possible (Bakken et al., 2011). If hindgut microbial colonization in early life would have long-term effects and influence production traits later in life, this area requires further investigation.

In this experiment, we studied six monozygotic twin-calf pairs that we recruited for an early-life rumen modulation study. Starting from the 2nd week after birth to the end of the pre-weaning period, one calf from each pair was given an oral dose of adult cow rumen liquid. The results demonstrated that the inoculum stimulated earlier maturation of rumen bacterial ecosystem in treated calves before and after weaning, with additional positive effect observed on the growth in young animals (Huuki et al., 2022a,b). Later, the same animals were followed up during their first lactation period, where differences between the groups were observed in residual energy intake and milk yield at different phases of lactation among others (Huuki et al., 2022b). As the colonization routes of the rumen and hindgut are interconnected, our next objective was to elucidate if early-life rumen microbiota modulation also affected the colonization and development of the colonic microbiota or had beneficial effect on gut health in young animals. Therefore, we explored the possible colonization routes associated with the colostrum, own rumen, or donor inoculum of microbes into the large intestine. We hypothesized that the inoculum treatment could also affect the large intestine microbiota and improve the gut health.

## Materials and methods

### Animals and experimental design

Six pairs of identical twin calves (four female and two male pairs) were produced by embryo splitting technique at Natural Resources Institute Finland (Luke). At birth, calves were randomly assigned to either a treatment (T-group) group or a control (C-group) group and separated into individual pens (147 cm × 172 cm) for the whole pre-weaning period to avoid direct contact between the calves or with other animals. Within 2 h of birth, all the calves received 2 L of the same high-quality (Brix value 24) colostrum mix, collected in advance from 3 cows, and stored at −20°C. As a second meal, the calves were offered colostrum from their own mothers, and thereafter, bulk colostrum was offered. Management and feeding of animals during the pre-weaning period are described in detail by Huuki et al. (2022a). During weeks 2 and 3, the T-group calves were given 5 mL of fresh rumen liquid orally, which was obtained from a feed efficient adult cow (Mäntysaari et al., 2012). During weeks 4–8, the dose was increased to 10 mL. Inoculation was performed weekly on Mondays, Wednesdays, and Fridays and is described by Huuki et al. (2022a). The C-group calves remained untreated but otherwise were managed and fed as the T-group animals. All calves were weaned at the age of 8 weeks, and the animals from both the T-group and C-group were placed into a group pen and housed together first at the Natural Resources Institute Finland (Luke) research barn and later in VikingGenetics breeding facility in Hollola, Finland. The bull calves were removed from the experiment after weaning. Management and feeding of heifers during the post-weaning period are described in detail by Huuki et al. (2022b).

### Health

The general health was monitored daily, and diarrhea cases in young animals were recorded by the barn veterinarian. During the pre-weaning period, four calves had loose feces and were treated with electrolyte supplement (Benfital Plus nutrient supplement, Boehringer Ingelheim Danmark A/S), or in more severe cases, three calves were given coal paste (Lehmän HIILI-pasta, FinnCow, Finland). For the analysis of microbial community changes, a calf was recorded to have diarrhea if symptoms occurred within ±2 days around the fecal sample collection time.

### Sample collection

Aliquots of the colostrum mix, provided as the first meal, and colostrum from each twin pair’s mother, given as the second meal, were collected into sterile 2 mL screw cap tubes and snap-frozen in dry ice. Fecal samples from calves were collected directly from rectum once a week when the calves were 1–8 weeks old. After weaning, the fecal samples were collected monthly starting from month 4 until animals reached 12 months of age. Feces, either freshly excreted or directly from the rectum, were collected before feeding at 1000–1100 h and immediately placed into sterile bags and snap-frozen in dry ice. Rumen samples from calves were collected at the age of 2, 4, 6, and 8 weeks via esophageal polyvinyl chloride tube (9/13 inner/outer diameter) with a vacuum pump (Ruminator, Germany) and from the donor animal though the fistula as described by Huuki et al. (2022a). All samples were stored at −80°C until DNA extraction.

### DNA extraction and sequencing

The total DNA was extracted from 150–250 mg of feces following the protocol by Yu and Morrison (2004) for samples collected during weeks 1–4 and as described by Rius et al. (2012) for samples collected from week 5 to adulthood. Both methods included bead beating step using MP FastPrep-24 (MP Biomedicals, USA). The total DNA extraction from rumen samples was described by Huuki et al. (2022a), while the DNA from 1 mL of colostrum samples was extracted using Milk Bacterial DNA Isolation Kit (Norgen Biotek Corporation, Ontario, Canada), following the manufacturers protocol. Bacteria and archaea were determined by primers 515F and 806R (Caporaso et al., 2011) targeting the 16S rRNA gene V4 region, while for fecal anaerobic fungi identification, primers Neo 18SF and Neo5.8SR targeting the ITS1 region (Edwards et al., 2008) were used. The sequencing libraries were prepared as described by Huuki et al. (2022a) and sequenced in Finnish Functional Genomics Centre (Turku, Finland) on Illumina MiSeq platform by using 2 × 250 bp chemistry for bacteria and archaea and 2 × 300 bp for anaerobic fungi.

### Sequence data processing

The demultiplexed bacterial and archaeal sequencing data were processed using QIIME 2 v2022.2 (Bolyen et al., 2019). The DADA2 (Callahan et al., 2016) workflow was applied for quality control, filtering of chimeric reads, and clustering of bacterial sequences into amplicon sequence variants (ASVs). The ASVs with less than 10 reads were removed. Silva 138 database (Quast et al., 2013) was used for bacterial taxonomy assignment and the RIM-DB database (Seedorf et al., 2014) was used for the archaeal taxonomy assignment. The anaerobic fungi sequencing data were processed with QIIME 1.9.1 (Caporaso et al., 2010) as described by Huuki et al. (2022a), and fungal taxonomy was assigned using the ITS1 (Koetschan et al., 2014) reference database. After removal of low abundance reads, the data consisted of 2,989,290 bacterial, 22,303 archaeal, and 4,569,340 fungal reads. The raw sequence reads are available in the NCBI Sequence Read Archive under BioProject PRJNA713003 with BioSample accessions SAMN36868668-36868795 and SAMN36903992-36904023. The anaerobic fungi mock communities, used as controls for sequencing, showed bias toward *Piromyces* sp. strain being assigned to *BlackRhino* clade. All *BlackRhino* clade OTUs and related reference sequence were compared against the NCBI database with BLASTN (Zhang et al., 2000).

### Quantitation of microbial communities

The quantities of bacteria, archaea, and anaerobic fungi were estimated with qPCR by quantifying rRNA/ITS1 gene copy numbers of each taxonomic group in 1 ng of extracted DNA. The quantitation was performed as described by Huuki et al. (2022b).

### Statistical analyses

Differences in microbial quantities, measured as gene amplicon copy numbers, were estimated using Linear Mixed Model GLIMMIX in SAS 9.4 (SAS Institute Inc., Cary, NC, United States). The bacteria copy numbers were log10(x) transformed for normality. Treatment, age, and the treatment × age interaction were treated as fixed effects. Pair was treated as random while age was treated as a repeated effect for each animal. Based on fit statistics, the covariance structure was set to compound symmetry for bacteria and spatial power for fungi and archaea. The difference in variance between groups was considered in bacteria and fungi.

Alpha diversity was estimated using Shannon and Simpson indexes, and the richness (number of observed ASVs) as implemented in *Phyloseq* (McMurdie and Holmes, 2013). Before analysis, the sequencing data were evenly subsampled to 3,187 reads for bacteria, 11,688 reads for anaerobic fungi, and 100 reads for archaea. The statistical analysis was performed using linear mixed model with fixed effects as described above and compound symmetry covariance structure. To achieve the normal distribution of residuals, the Simpson index was (x)^2^ transformed for bacteria and anaerobic fungi, and least square means were later reverse transformed. The effects were estimated using the Residual Maximum Likelihood (REML) method and were declared significant at *p* ≤ 0.05. Due to the low number of reads for archaea, the statistical analysis was performed only on samples from month 4 onward.

Changes in microbial community structure between samples were evaluated as Bray–Curtis dissimilarities following Hellinger transformation and visualized using principal coordinate analysis (PCoA) with *Phyloseq*. The significance of age, treatment, and treatment x age interaction was estimated using distance-based permutational multivariate analysis (Adonis) as implemented in *vegan* (Oksanen et al., 2022) and the pairwise comparisons between age × treatment with permutational multivariate analysis of variance with false discovery rate adjustment with *RVAideMemoire* (Herve, 2020).

Age- and group-related taxonomic differences were evaluated at phylum and genus level for bacteria and at species level for archaea and anaerobic fungi. Samples with less than 10 archaeal reads were excluded from the analysis. All data were log(1 + x) transformed for normalization. The significance of age and treatment × age interaction was evaluated using ANOVA.

The microbiota differences between the healthy calves and the calves experiencing diarrhea symptoms were identified with the linear discriminant effect size (LEfSe) analysis, with logarithmic LDA score of 2.0 for discriminative features and 0.05 alpha value for both factorial test among classes (healthy, diarrhea) and pairwise test for subclasses (T-group, C-group) (Segata et al., 2011). Before analysis, the ASVs that were observed in less than 4 samples were removed.

The SourceTracker version 1.0.1 (Knights et al., 2011) was used to assess the possible seeding sources of hindgut prokaryote ASVs (bacteria and archaea) and fungal OTUs. For prokaryote seeding, colostrum samples from twin calves’ mothers, calves’ rumen samples from weeks 2, 4, 6, and 8, and the inoculum samples from donor rumen were treated as sources. The fecal samples from week 1 to week 8 were treated as sinks. For hindgut anaerobic fungi seeding, the rumen samples from months 4–9 and the donor samples were treated as sources, with fecal samples from the same time period were treated as sinks. For the analysis, the core ASVs/OTUs for the rumen, colostrum, and donor inoculum samples were shortlisted for each time-point separately with detection rate of at least 70% and relative abundance of above 0.001% for bacteria and 0.1% for anaerobic fungi.

## Results

### Bacteria

**Figure 1:**
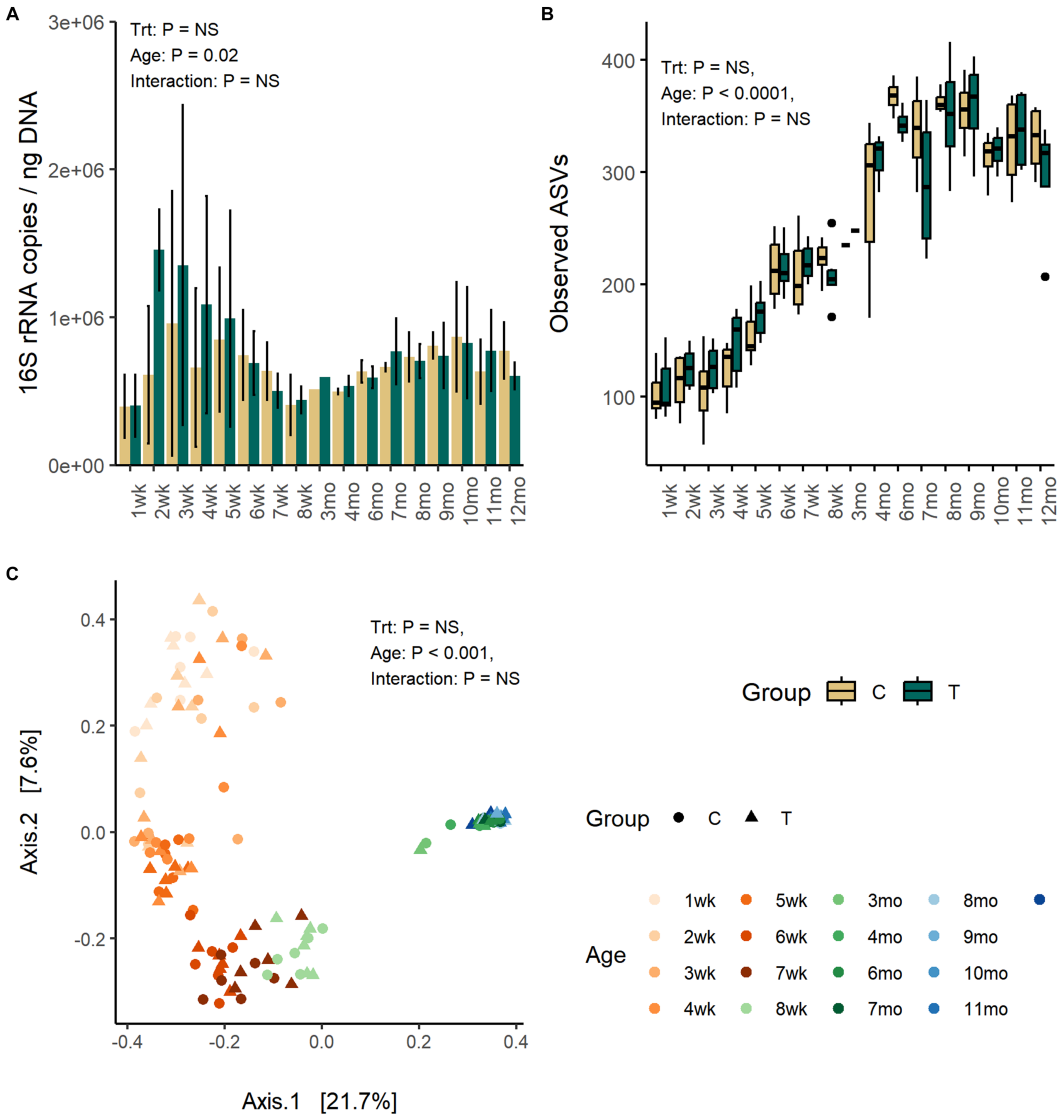
The number of bacteria 16S rRNA gene copies per ng of extracted DNA **(A)**, the number of observed ASVs **(B)**, and the PCoA visualization of Bray–Curtis distances **(C)** of fecal bacteria communities in rumen liquid inoculated (T) and control animals (C) from 1 weeks to 12 months age.

The quantity of fecal bacteria in the T-group between weeks 2 and 4 was numerically higher as compared with C-group, but the differences between both groups at the same time-points were not significant (Figure 1A). The fecal bacterial load changed with age (*p* = 0.02). The copy-numbers were low at week 1 but increased in quantity right after and reached a peak between weeks 2 and 3. After that, a decline in bacterial quantity was observed until week 8 when the calves were weaned. Post-weaning quantity in fecal bacteria increased moderately and stabilized in approximately 10 months of age.

Fecal alpha diversity was significantly affected by age but not by inoculum treatment or interaction between treatment and age. The number of observed ASVs and Shannon diversity was increased with age (*p* < 0.0001) and was highest between months 6 and 9 and thereafter stabilized (Figure 1B; Supplementary Figure S1).

The PCoA visualization of Bray–Curtis distances revealed large between-animal variation in fecal bacterial community composition at weeks 1 and 2, which shifted to the first intermediate state for the weeks 3–5 and second state for weeks 6–8, finally becoming mature and uniform after month 3 (Adonis: *p* = 0.001) (Figure 1C). The changes in community composition likely reflected the dietary changes from milk only to increasing amount of solid feed towards increasing starter concentrate diet and later introduction of silage. The treatment (Adonis: *p* = 0.26) or interaction between treatment and age (Adonis: *p* = 0.86) had no effect on the community composition.

Firmicutes (57–87%) and Bacteroidota (9–31%) were the predominant fecal phyla at all time-points (Supplementary Figure S2). Proteobacteria, mainly dominated by *Escherichia-Shigella* (4–11%), and Actinobacteriota (genus *Bifidobacterium*) (2–12%) were present in young animals but reduced in abundance toward weaning and diminished to <1% abundance in heifers (*p* < 0.0001). Verrucomicrobiota (*Akkermansia* and WCHB1-41) (0–3%) was increased in abundance with age (*p* < 0.0001), in particular after month 4. *Akkermansia* was numerically more abundant in the C-group at approximately week 8 (Treatment: *p* = 0.07).

**Figure 2:**
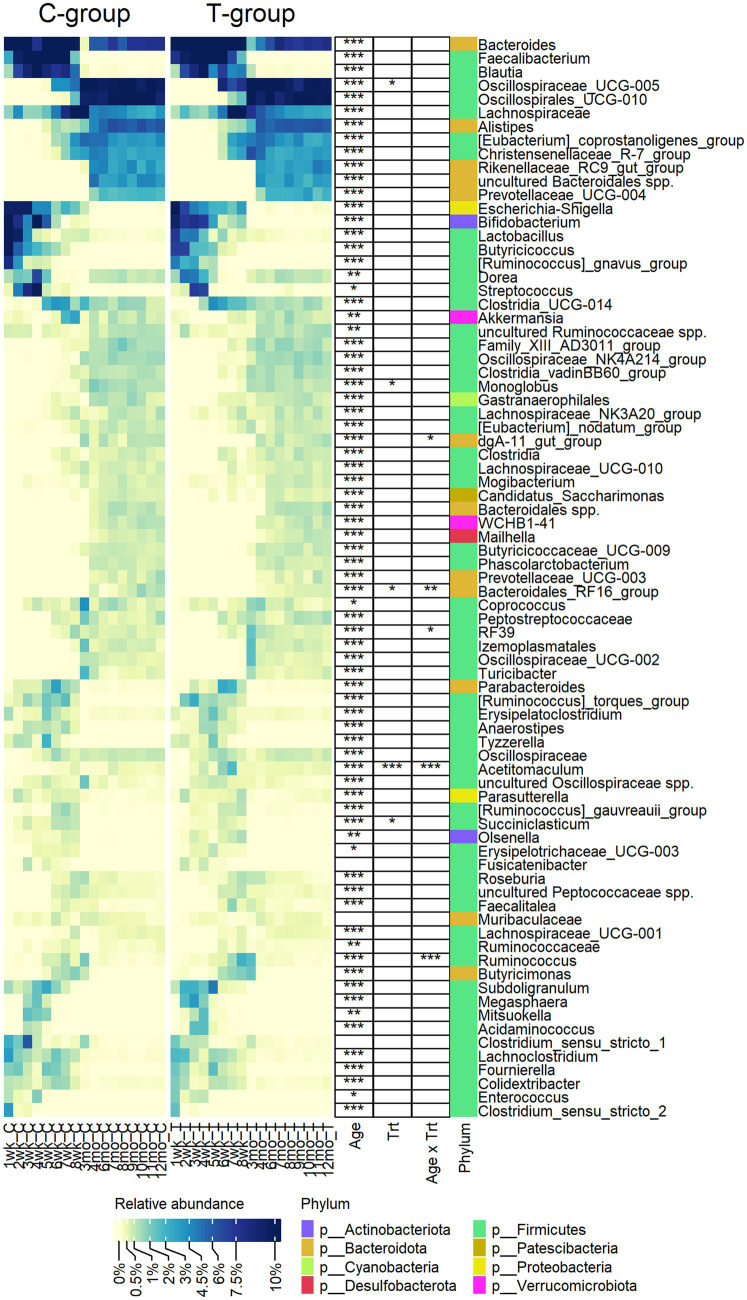
The relative abundance of bacterial genera observed in the feces of rumen liquid inoculated (T) and control (C) animals from week 1 to 12 months of age. The statistically significant effects of age (Age), treatment (Trt), and age x treatment interaction (Age x Trt). The colour bar indicates the affiliation to phylum.

Out of the 79 genera, with relative abundance above 0.2%, 76 were affected by age and categorized fecal bacterial community into pre-weaning and post-weaning genera (Figure 2). *Bacteroides, Lachnospiraceae* spp.*, Dorea, Ruminococcaceae* spp.*, Akkermansia*, and *Coprococcus* were among the few genera present in feces throughout the first year of life, although their abundance varied with age. While calves were fed liquid diet (weeks 1–4), feces was dominated by a small number of higher abundant taxa, including *Bifidobacterium* (Actinobacteriota), *Bacteroides* (Bacteroidota), *Lactobacillus*, *Streptococcus*, *Dorea*, *Blautia*, *Faecalibacterium*, *Butyricoccus*, *Ruminococcus gnavus* group (Firmicutes), *Escherichia-Shigella* (Proteobacteria), and several lower abundant genera representing Firmicutes. When gradual weaning started at week 5, most of the taxa observed earlier remained present. In addition, *Alistipes*, *Parabacteroides*, *Butyricimonas*, *Oscillospiraceae* UCG-005, *Christensenellaceae* R-7 group, or [*Eubacterium*] *coprostanoligenes* group appeared at abundance above 1%. The biggest change was visible after weaning starting from month 3, where pre-weaning-related taxa were replaced at different time-points by 34 genera, which were associated with maturation of the fecal bacterial community. Post-weaning fecal bacterial community was composed of many more taxa, as compared with early life samples, each sharing low to moderate (0.5–5%) abundance. There was an increase in diversity of Bacteroidota, where *Rikenellaceae RC9* gut group, *Prevotellaceae* UCG-004, and *Bacteroidales RF16* group that were not detected in early life samples became abundant. Among Firmicutes, a different process was observed. Many pre-weaning-related Firmicutes genera were diminished in abundance or were not anymore detected in post-weaning samples (members of Negativicutes class or genera such as *Faecalibacterium*, *Butyricoccus*, *Fournierella*, *Subdoligranulum*, [*Ruminococcus*] *gnavus group, and Enterococcus*). Instead, several other genera belonging to Oscillospirales, Peptostreptococcales-Tissierellales, or Christensenellales orders increased in abundance when animals reached adulthood (Figure 2).

Eight genera were significantly affected by treatment or interaction between age and treatment (Figure 2). The T-group had an overall lower abundance of *Succiniclasticum* (*p* = 0.013), *Monoglobus* (*p* = 0.05), and *Bacteroidales* RF16 group but significantly more abundance of *Oscillospiraceae* UCG-005. Other differences between the groups were related to temporary fluctuation in the abundance of several genera, e.g., *Acetitomaculum* was more abundant in the T-group between weeks 4 and 8 (Interaction: *p* < 0.0001), while *Ruminococcus* was significantly higher (*p* < 0.0001) and *RF39* was lower (pairwise: *p* = 0.003) in T-group on week 8.

### Archaea

**Figure 3:**
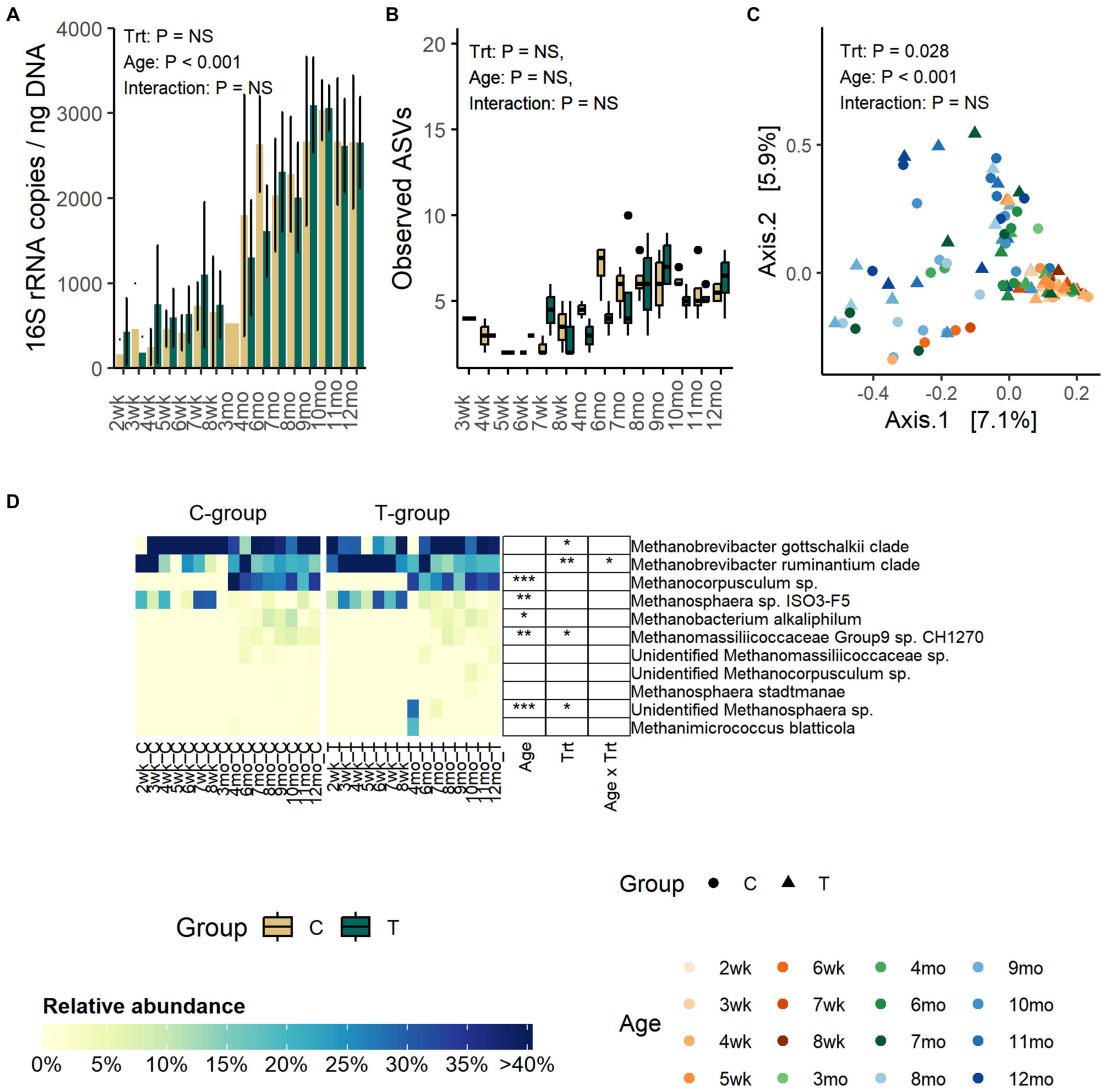
The number of archaea 16S rRNA gene copies per ng of extracted DNA **(A)**, the number of observed ASVs **(B)**, the PCoA visualization of Bray–Curtis distances of archaea communities **(C)** and the relative abundance of fecal archaea species **(D)** in rumen liquid inoculated (T) and control animals (C) from week 1 to 12 months of age. In panel **(B)** statistical analysis was performed for months 4–12 samples only due to low number of reads and missing observations in pre-weaning samples. For alpha diversity calculation, archaea data were evenly subsampled, and samples with less than 100 reads were removed from the analysis.

The quantity of fecal archaea was low until month 4, indicating that the community started to properly develop only after weaning (Figure 3A). The copy numbers increased until month 8, after which they stabilized (*p* < 0.001). Treatment or interaction between treatment and age did not affect the quantity of archaea (Treatment: *p* = 0.99, Interaction: *p* = 0.27).

Alpha diversity was not affected by treatment or interaction between treatment and age (Figure 3B; Supplementary Figure S3). Numerically, richness remained low until month 4 after which the number of observed ASVs started to increase and reached the peak in the C-group on month 6 and in the T-group on month 9. After month 9, the observed ASVs decreased and plateaued.

The PCoA visualization of Bray–Curtis distances showed that the pre-weaning archaeal community composition was very similar between individuals, but after weaning the variation greatly increased (Adonis: Age: *p* = 0.001) (Figure 3C).

Before month 4, the fecal archaeal community was dominated by *Methanobrevibacter gottschalkii* clade (17–100%), *Mbb. ruminantium* clade (7–92%), and *Methanosphaera* sp. *ISO3-F5* (4–32%) (Figure 3D). From month 4 onwards, the community experienced changes. *Mbb. gottschalkii* and *Mbb. ruminantium* clades continued as the most abundant species, while *Methanosphaera* sp. *ISO3-F5* abundance diminished. Instead, *Methanocorpusculum* sp., which was not detected in pre-weaning samples, increased in abundance (16 – 41%) (Age: *p* < 0.001). *Methanobacterium alkaliphilum* and *Methanomassiliicoccaceae Group 9* sp. were detected in fecal samples in month 6, and they remained in the community thereafter.

In comparison to the C-group, the T-group had a higher overall abundance of *Mbb. ruminantium* clade (Treatment: *p* = 0.007) and lower abundance of *Mbb. gottschalkii* clade, with differences apparent between months 1 and 3. During the post-weaning period, the T-group had less of *Methanomassiliicoccaceae Group9* sp. *CH1270* (Treatment: *p* = 0.045). Two calves in the T-group were observed to have *Methanosphaera* sp., which was not observed in any of the C-group calves (Treatment: *p* = 0.02; Interaction: *p* < 0.0001).

### Anaerobic fungi

**Figure 4:**
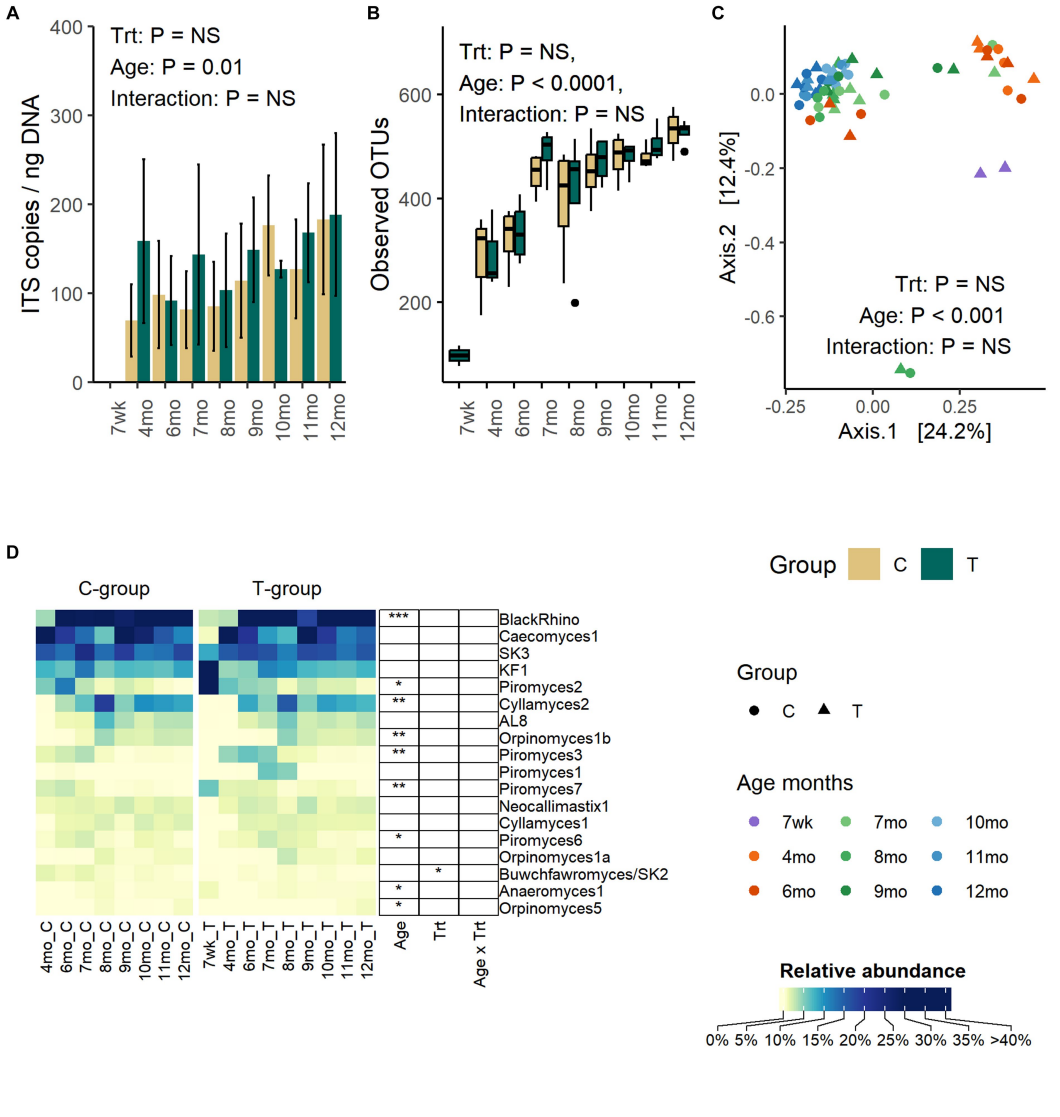
The number of anaerobic fungi ITS1 gene copies per ng of extracted DNA **(A)**, the number of observed OTUs **(B)**, the PCoA visualization of Bray – Curtis distances **(C)**, and the average relative abundance of anaerobic fungi in the feces of rumen liquid inoculated (T-group) and control (C-group) animals between week 7 to month 12 of age (D). The qPCR method could only reliably detect anaerobic fungi after month 4, although some sequencing data could be obtained from 2 T-group samples at week 7. The data for samples at week 7 are visualized in the figure but excluded from the statistical analysis.

Anaerobic fungi could be reliably detected in fecal samples by the qPCR method only after weaning. Between months 4 and 12, the copy numbers were significantly increased (Age: *p* = 0.01) but were not affected by treatment (Treatment: *p* = 0.47, Interaction: *p* = 0. 68) (Figure 4A).

The number of observed OTUs was increased significantly with age until month 8 after which the richness stabilized (Age: *p* < 0.0001) (Figure 4B). Similarly, Shannon diversity (Age: *p* < 0.0001) and Simpsons evenness (Age: *p* < 0.0001) increased until month 7 and then stabilized (Supplementary Figure S4). Treatment or interaction between treatment and age had no significant effect on any of the alpha diversity metrices.

The age, but not treatment, affected the fecal fungal community composition (Adonis: Treatment: *p* = 0.65, Age: *p* = 0.001, Interaction: *p* = 1.0) (Figure 4C). The PCoA visualization of Bray–Curtis distance categorized communities into clusters based on age, where samples between week 7 and month 4 grouped together. Between months 6 and 9, communities were transitioned into a more mature state, but the composition varied between individual heifers. At months 9–12, the fungal communities of all heifers were similar and grouped together.

Although pre-weaning samples exhibited low amplification success with other PCR methods, the sequencing of ITS1 libraries from two T-group calf samples proved successful on week 7. Because of the low observation rate and low number of reads, these samples were excluded from the statistical analyses, but the taxonomic composition is visualized (Figure 4D). The week 7 samples differed from later samples, predominantly being composed of *Piromyces 2* (44%) and *KF1* (38%). By month 4, *KF1* and *Piromyces 2* groups had decreased by more than six folds, and the fecal fungal community was dominated by *Caecomyces 1* (63%), *SK3* (16%), and five other less abundant species (0.4–3.5%). On month 6, the *BlackRhino* became the most abundant clade (33%), which was accompanied by five other abundant (5.5–20%) species-level clades: *Caecomyces 1*, *SK3*, *Piromyces 2*, *Cyllamyces 2*, and *KF1*. All *BlackRhino* OTUs in our data set were associated with one reference ITS1 sequence (MH039104) that showed over 99% identity to the same *Piromyces* sp. SFH682 strain, which has been previously shown to be identical to *BlackRhino* (Fliegerova et al., 2021). On month 7, the community maintained *BlackRhino*, *Caecomyces 1*, *SK3*, *Cyllamyces 2*, and *KF1* as the top five most abundant species, while *Piromyces 2* started to decline, and this trend remained the same until month 12. The highest species-level richness (18 species level taxa) was observed by month 8. Between months 8 and 9, *Cyllamyces* 2. *Orpinomyces* spp., and AL8 experienced fluctuation in relative abundance, but between months 10 and 12, the community composition stabilized. On month 12, all *Piromyces* spp. had reduced below 1% relative abundance. Taking into consideration the relatedness of *BlackRhino* to *Piromyces* species, the results indicate that different *Piromyces* spp. were important at early and late post-weaning stages. The T-group had an overall higher abundance of *Buwchfawromyces/SK2* species (*p* = 0.023), but the abundance was not affected by age or interaction between treatment and age.

### Gut health

**Figure 5:**
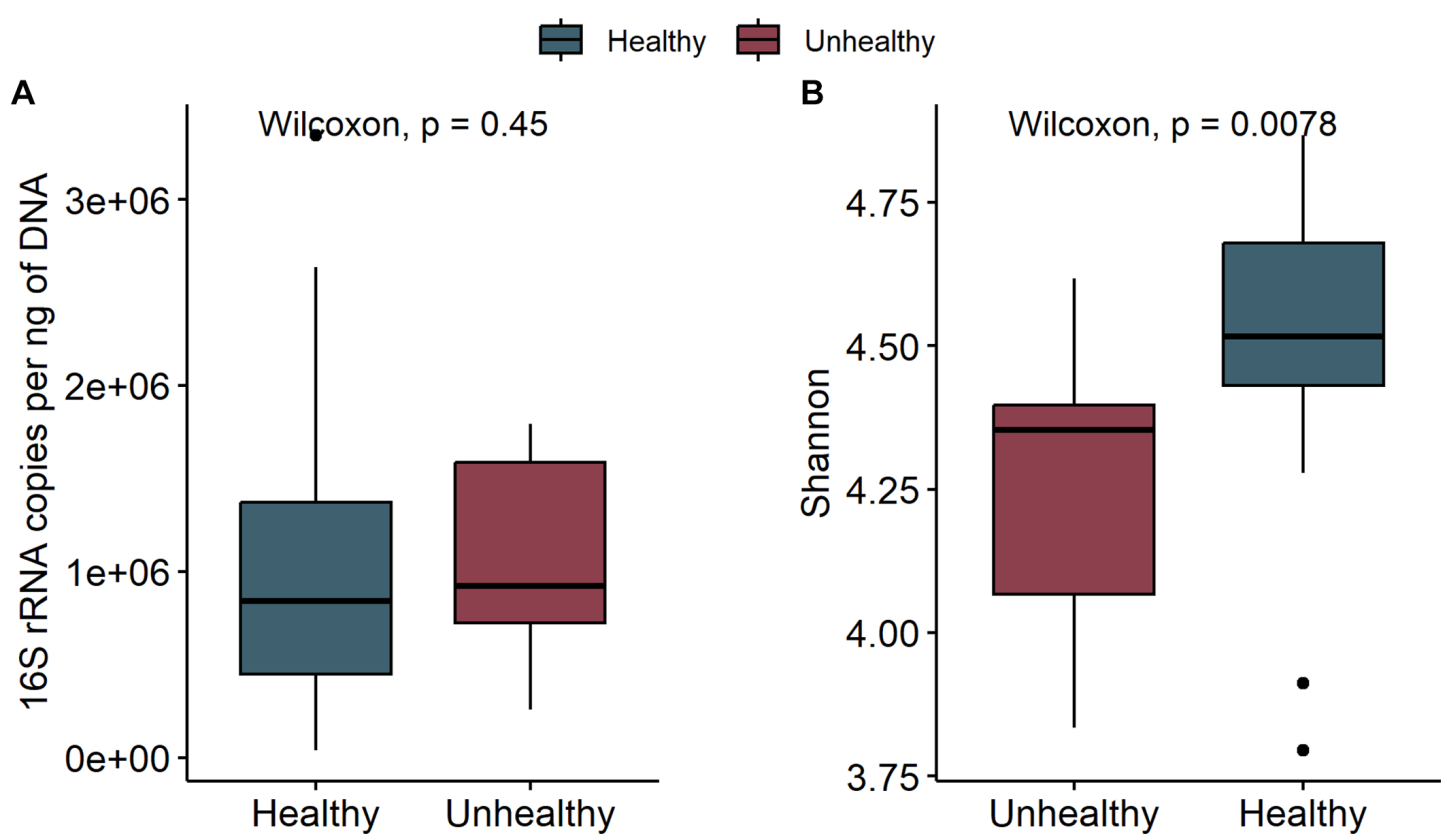
The comparison of bacteria 16S rRNA gene copies per ng DNA **(A)** and the Shannon diversity **(B)** of fecal samples in healthy calves (healthy) and calves with observed diarrhea (unhealthy) at 2 to 4 weeks of age.

**Figure 6:**
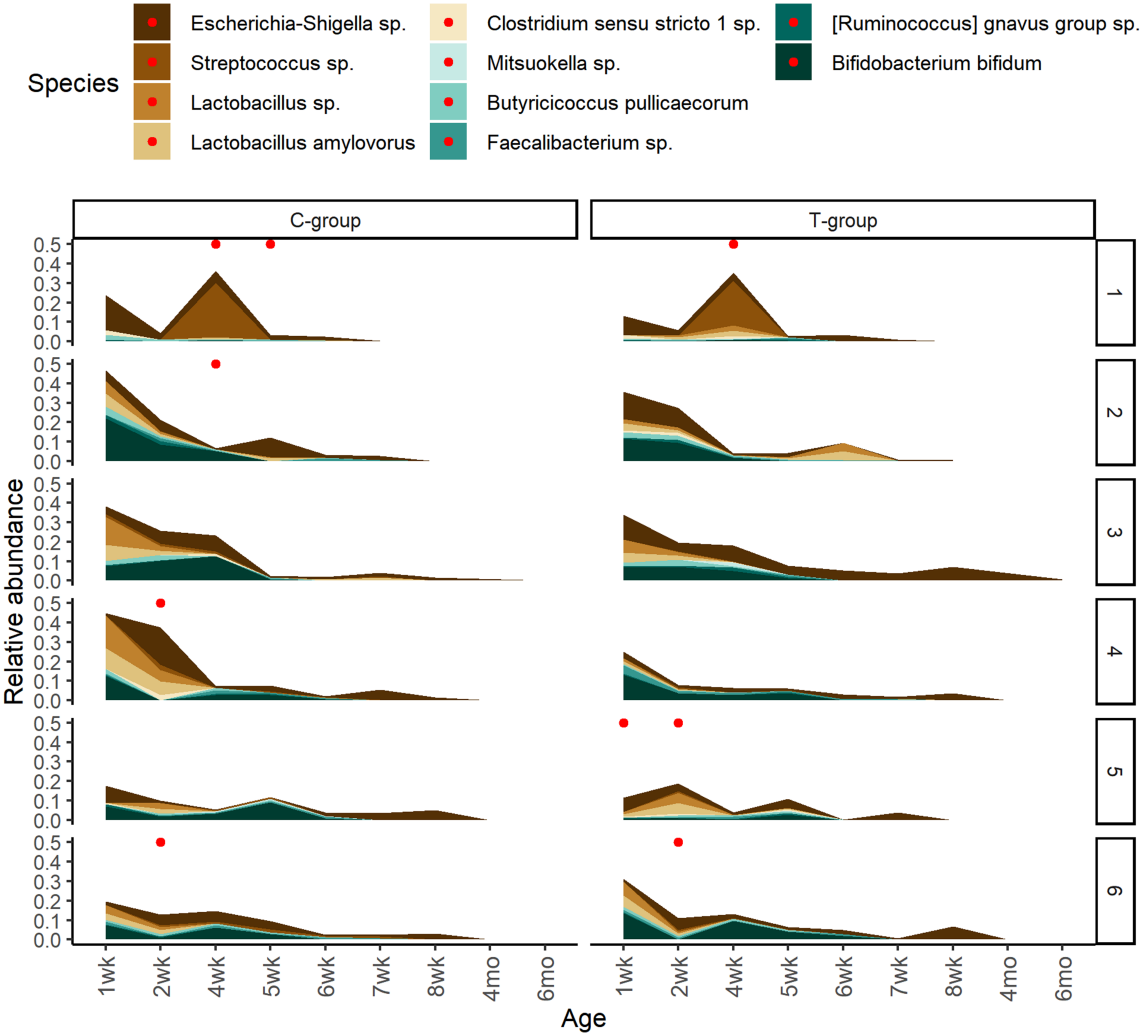
The temporary changes in the relative abundances of differently abundant bacterial ASVs between healthy calves and calves with observed diarrhea cases grouped at species level. The reported diarrhea occurrences (within +/− 2 days from sampling day) are indicated with red dots.

To assess the potential effect of inoculum treatment on gut health in pre-weaning animals, we examined microbiota differences between healthy calves and calves with reported diarrhea symptoms. Five of the C-group calves and four of the T-group calves were observed to have diarrhea between 2 and 4 weeks of age. The diarrhea did not affect the bacteria quantity (Figure 5A) but significantly reduced the alpha diversity (Figure 5B). The LEfSe analysis identified 34 differentially abundant ASVs between healthy and diarrhea samples, which were affiliated with 10 genus- or species-level taxa (Supplementary Figure S5; Figure 6). Fecal microbiota in healthy calves between weeks 2 and 4 exhibited higher abundance of ASVs associated with *Ruminococcus gnavus* group sp., *Bifidobacterium bifidum*, *Butyricicoccus pullicaecorum*, and *Faecalibacterium* sp., while fecal microbiota of diarrhea samples was characterized by higher abundance of ASVs associated with *Escherichia-Shigella* spp.*, Streptococcus* spp.*, Clostridium sensu stricto* 1 sp., and *Lactobacillus* spp. None of the differentially abundant ASVs were significantly associated with the donor inoculum treatment. In all individuals, taxa that differentiated between healthy and diarrhea cases in early life disappeared from the fecal community by month 6 (Figure 6).

### Potential seeding sources of the fecal bacteria

**Figure 7:**
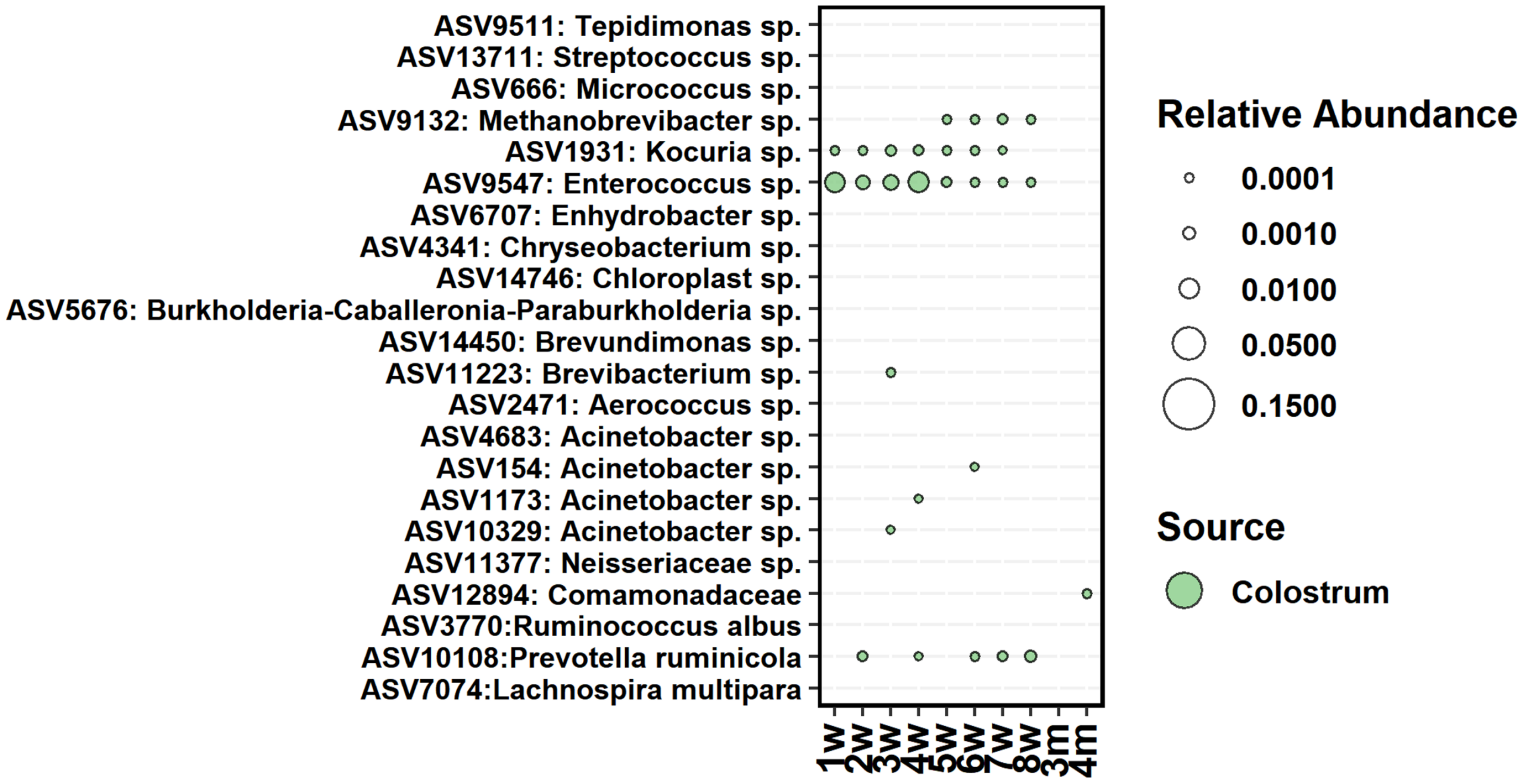
The average relative abundance of colostrum core ASVs detected in fecal samples of calves between week 1 and 4 months of age.

The cow colostrum demonstrated large between-sample variation in bacterial composition. Therefore, for seeding analyses, we shortlisted colostrum bacteria to 22 ASVs, present in 70% of samples (Figure 7). From them, only one *Enterococcus* sp. ASV was present in feces between weeks 1 and 8, *Kocuria* between weeks 1 and 7, and *Prevotella* between weeks 2 and 8. *Acinetobacter* was detected in feces shortly between weeks 3 and 6, while *Methanobrevibacter* was present in feces between weeks 5 and 8. All colostrum-derived ASVs were transient in feces, and none of them remained during the post-weaning period.

**Figure 8:**
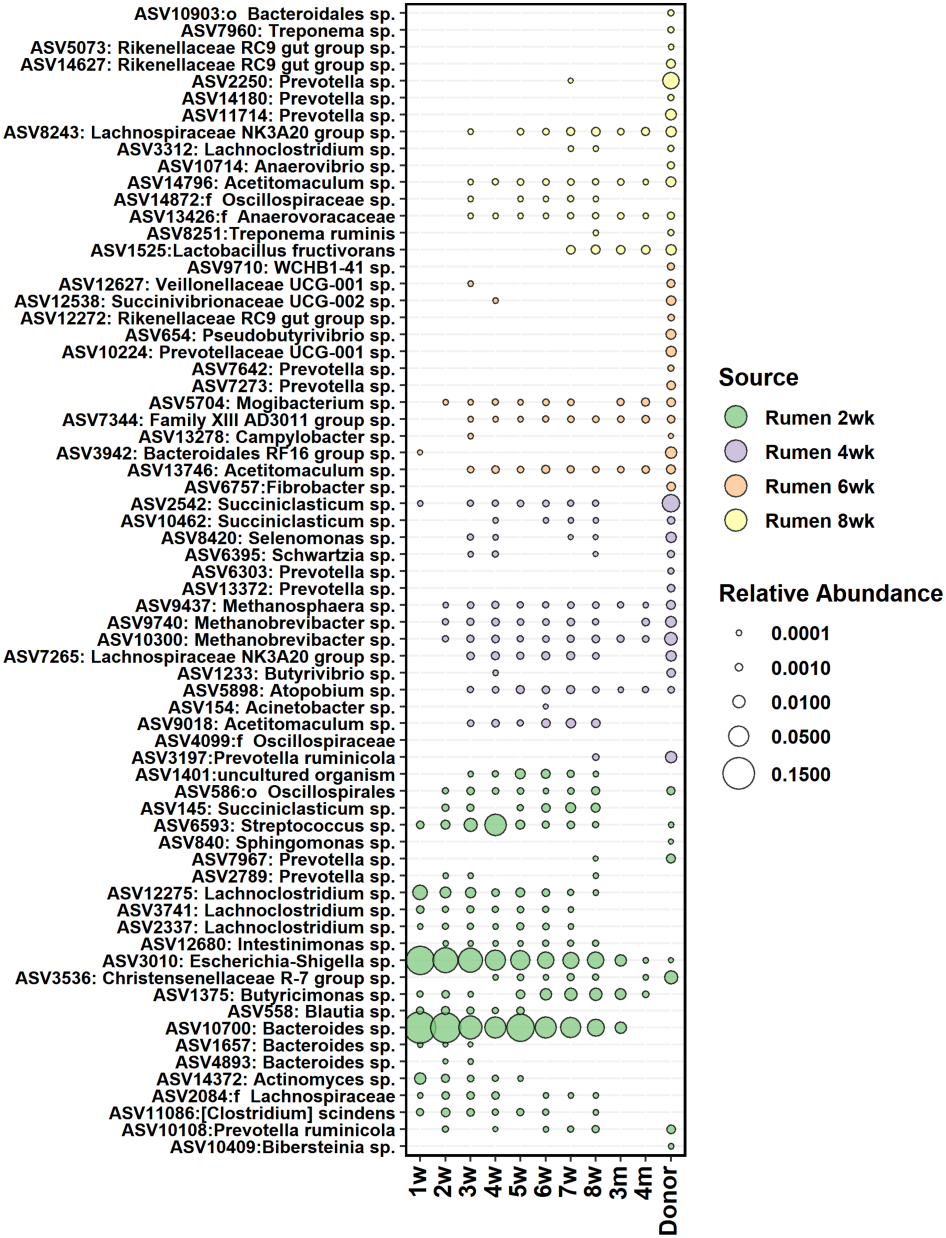
The average relative abundance of rumen core bacteria ASVs from week 2, 4, 6, and 8 detected in fecal samples of calves between week 1 and 4 months of age and in donor inoculum. The different colors indicate new ASVs introduced to the core community during each time-point.

The core bacteria from calf rumen shortlisted for seeding analysis was comprised of 23, 22, 21, and 25 ASVs on weeks 2, 4, 6, and 8, respectively. Most of the ASVs in the rumen core community of 2 weeks old calves were also detected in 2-week-old fecal samples (Figure 8) and showed 5–41% contribution to fecal bacterial colonization (Supplementary Figure S6). The majority of them were transient: *Actinomyces* and *Blautia* were present in feces until week 5, while *Prevotella*, *Streptococcus*, *Lachnoclostridium*, and *Succiniclasticum* disappeared from feces after week 8. *Bacteroides* was still detected in feces at month 3, and *Butyricimonas* was detected at month 4. On the other hand, *Escherichia-Shigella*, *Methanobrevibacter*, *Methanosphaera*, and *Mogibacterium* colonized early and remained in feces permanently. After week 2, the proportion of calf rumen core ASVs shared with feces decreased, suggesting that the rumen and large intestine started developing more specialized microbial ecosystems. *Acetitomaculum* and *Lachnospiraceae NK3A20* groups were transient in feces between weeks 3 and 8, while *Atopobium*, Family XIII AD3011 group, and *Christensenellaceae R-7* group populated at weeks 3–4 and remained in feces permanently, suggesting that these taxa might be needed for the initiation of solid feed digestion. Interestingly, ASV affiliated with *Lactobacillus fructivorans* that was not detected in the rumen before week 6 and in feces before week 7 remained in both ecosystems until month 12 and indicated an association with the transition to solid diets. Based on the SourceTracker analysis, donor inoculum was not identified as the major seeding source for fecal bacteria in calves (Supplementary Figure S6).

### Potential seeding sources of the fecal anaerobic fungi

**Figure 9:**
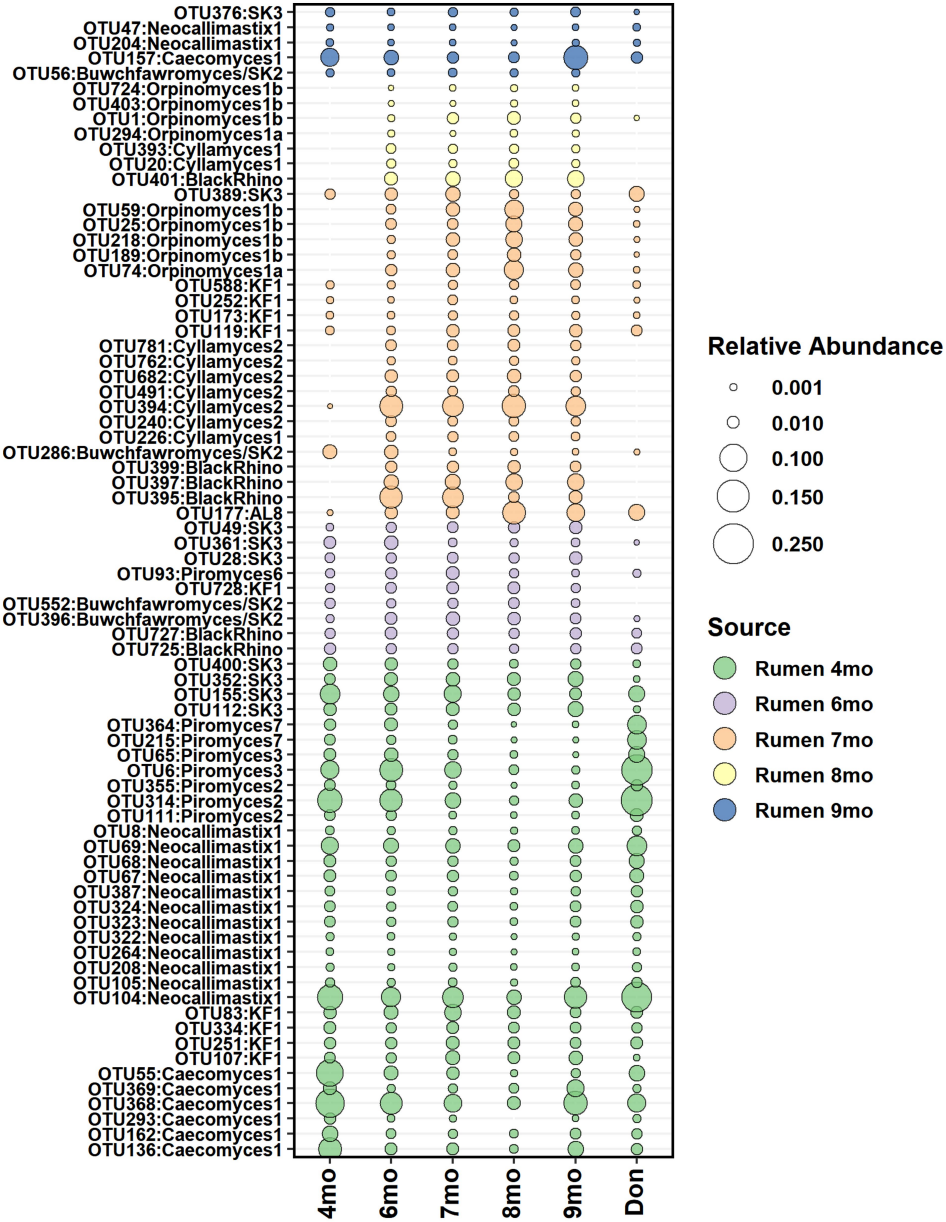
The average relative abundance of rumen core anaerobic fungi OTUs detected in fecal samples of heifers between month 4 and month 9 of age and in donor inoculum. The different colors indicate new OTUs introduced to the core community during each time-point.

According to the SourceTracker analysis, the calf’s own rumen was identified as the primary seeding source of anaerobic fungi in feces, accounting for 98.7, 68.5, 79.2, 94.0, and 95.6% of the fecal community composition between months 4 and 9, respectively (Supplementary Figure S7). The heifer rumen core anaerobic fungi OTUs shortlisted for seeding analysis comprised of 33, 32, 52, 36, and 59 OTUs on months 4–9, respectively. On months 4 and 6, all rumen OTUs of anaerobic fungi were consistently detected in feces and persisted in the community (Figure 9).

Donor inoculum did not show contribution as seeding source at month 4, as all rumen anaerobic fungi detected in heifers at month 4 were also observed in the donor. However, starting from month 6, several new rumen anaerobic fungi OTUs were observed in feces, and these were distinct from the donor community. The timing coincided with the transfer of calves to a different barn, where exposure and acquisition of new fungi from new barn environment was possible.

### Probiotic strains added to feed

The commercial milk replacer offered to calves contained *Bacillus licheniformis* (DSM 5749), *Bacillus subtilis* (DSM 5750), and *Enterococcus faecium* (NCIMB 11181). We tested if these species could be detected in fecal microbiota of calves. BLAST analysis of fecal *Enterococcus* ASVs against the NCBI database did not return 100% similarity match to given species. The closest 99.7% similarity was observed in *E. faecalis* and other uncultured *Enterococcus* sp., suggesting that the 16S rRNA V4 region is not able to separate between different species of *Enterococcus*. The one fecal ASV from the genus *Bacillus* did not match with either *B. subtilis* or *B. licheniformis*.

## Discussion

In this study, we examined the influence of early-life rumen microbiota modulation on the colonization and maturation of the fecal microbiota in dairy heifers over a 12-month period. We also explored modulation’s potential benefits for gut health in young animals. This research is an extension of our previous study, which demonstrated that dosing calves with fresh rumen liquid from an adult donor cow accelerated the maturation of rumen bacterial and archaeal communities, promoted the growth of calves (Huuki et al., 2022a,b), and impacted animal production performance during their 1st lactation (Huuki et al., 2022b). In the current study, we demonstrated that colostrum and the young animal rumen serve as sources for seeding the fecal bacterial and archaeal communities, while the donor rumen inoculum treatment had no impact on the composition of fecal microbiota. In addition, we provided new evidence that rumen also greatly contributes to hindgut anaerobic fungi colonization as a seeding source.

### Bacteria seeding sources and community development

Our results showed very distinct bacterial communities between pre-weaning and post-weaning samples, which coincided with the changes in the diet. After the first week, the bacterial load in feces quickly reached a stable state, but the community richness continued to increase until month 4. During the first 2 weeks, while the calves were fed milk diet, the fecal bacteria consisted mainly of *Bacteroides*, *Bifidobacterium*, *Faecalibacterium*, *Blautia*, *Lactobacillus*, and *Ruminococcus gnavus* group, well-known commensal bacteria. Many of them are known to have important roles in the developing gastrointestinal tract of calves (Liu et al., 2021; Gomez et al., 2022) and have been previously associated with large intestine microbiota in pre-weaning animals (Dias et al., 2018; Song et al., 2018). After the second week, when the milk diet was changed to milk replacer, *Lactobacillus* were replaced mainly by another facultative anaerobe *Streptococcus*. *Lactobacillus* is associated with milk digestion and is able to utilize lactose and starch for lactate production (Hammes and Hertel, 2015). In addition, *Streptococci* are able to utilize a variety of carbohydrates, e.g., lactose and starch, in fermentation (Whiley and Hardie, 2015). However, *in vitro* co-culturing of oral *Streptococcus* sp. and *Lactobacillus* sp. strains has shown pH-dependent competition among the genera (Bowden and Hamilton, 1989). The change in diet composition may have altered the available substrates and may have caused changes in hindgut fermentation, leading to altered pH, and caused the decrease in *Lactobacillus*.

As a seeding source, the colostrum’s contribution to the bacterial colonization in feces was temporary. Only *Enterococcus* sp., *Kocuria* sp., and *Prevotella ruminicola* were shared between colostrum and feces of calves during the pre-weaning period, suggesting the association of these taxa with the digestion of liquid diet. *Enterococci* are commonly found in the feces of pre-ruminant calves (Devriese et al., 1992) and are facultatively anaerobic, lactic acid-producing bacteria that are capable of utilizing various sugars (Švec and Devriese, 2015). *Kocuria* are typically associated with the skin microbial community and have been found among udder skin microbiota (Braem et al., 2013) and in milk of cows (Quigley et al., 2013; Shinozuka et al., 2021). The colostrum-related ASVs disappeared from the fecal community after weaning, which indicates that they had a functional role only during the pre-weaning period. Facultative anaerobes such as *Enterococcus* play a crucial role in creating and maintaining the anaerobic environment, which enable the colonization and function of obligate anaerobes. Therefore, we propose that colostrum-related taxa in fecal samples may serve a similar role in maintaining an anaerobic environment and digesting milk or liquid feed. Our results are in line with previous studies (Yeoman et al., 2018; Klein-Jöbstl et al., 2019) that colostrum acts as a seeding source for some important hindgut bacteria, but it also plays an important role in providing immunoglobulins and nutrients to host and microbes and thereby indirectly affects the community structure.

When calves’ own rumen microbiota was evaluated as a seeding source, an especially strong influx from the rumen into feces was observed at weeks 2–4, and these taxa mostly remained in feces only until animals shifted to solid diets. To be able to colonize the large intestine, bacteria have to have suitable nutrients for growth, and they have to be able to withstand the acidic conditions in the abomasum and the bile acids and digestive enzymes. Our observations suggest that at an early age, when the rumen environment still resembles more than that of the hindgut, rumen serves as a reservoir for certain bacteria, which can transfer to and establish in the hindgut. However, after week 4, only a few new bacteria were introduced to the fecal community from the rumen likely due to functional specialization of both the rumen and hindgut microbial communities. The bacteria in the large intestine (like *Bacteroides*) were benefited by relying more on the host mucosa-derived glycans and xylans and cross-feeding of their breakdown products (Lin et al., 2023). Silage was introduced to the diet on 7th week, and by week 8, a small seeding from the more mature rumen community was observed. This was likely caused by the introduction of new substrates that provided new niches into the hindgut environment.

After week 4, when the gradual increase in solid feed intake started, *Bacteroides*, *Faecalibacterium*, and *Blautia* remained abundant, while *Bifidobacterium* decreased in abundance. *Bifidobacterium* are specialized in utilizing complex milk and mucus oligosaccharides (Marcobal et al., 2010; Zhang et al., 2022), while *Bacteroides* and *Blautia* are capable of not only utilizing milk and host-derived oligosaccharides and glycoconjugates (Song et al., 2015; Liu et al., 2021) but also can utilize a wider range of plant carbohydrates (Singh, 2019). The dietary transition was also accompanied by an increase in the abundance of many fiber-degrading Firmicutes (e.g., *Oscillospiraceae* UCG-005, *Lachnospiraceae*, and *Christensenellaceae* R-7 group). The rumen enzymatic activity reaches a stable state only after 1 month of age (Rey et al., 2012), and the starch digestion of the small intestine is not yet very efficient in calves (Gilbert et al., 2015). Increased flow of solid feed digesta into the large intestine may have benefitted these bacteria.

In comparison to the rumen bacterial community that matured at approximately 6 months of age (Huuki et al., 2022b), we found that the maturation of fecal bacterial community occurred earlier at approximately month 4. The community richness greatly increased, and the remaining pre-weaning related taxa (e.g., *Faecalibacterium* and *Blautia*) reduced and were replaced by several Firmicutes and Bacteroidetes genera, like *Rikenellaceae* spp. and *Prevotellaceae* spp., with broader capability to utilize plant carbohydrates. The earlier maturation of fecal bacteria community may be due to more stable digesta consistency and environment in the large intestine. When animals shift to solid diets and rumen fermentation becomes the major source of energy, the composition and flow of digesta into the hindgut becomes fairly constant (Constable et al., 2005, 2006). On the contrary, in the rumen, the change in volume size and development of muscle mass still continues while animals grow during the post-weaning period. The diurnal variation between ingestion, rumination, and resting bouts or adjustment to changing passage rate may require more time for the rumen bacterial community to mature. Moreover, the digesta retention time is shorter, and digesta substrate consistency in the large intestine is less complex in comparison to the ingested diet in the rumen, as the complex dietary carbohydrates have undergone digestion by ruminal microbial enzymes and gastric enzymes in the abomasum and small intestine. Therefore, the hindgut bacteria community can probably cope with a more limited functional capacity, and the community is therefore able to mature earlier.

### Archaea seeding sources and community development

Significantly less information is available regarding methanogenesis and archaea community composition in the hindgut of ruminants compared with the rumen. The archaeal load in feces was lower before weaning than after weaning, indicating that the proper community establishment occurred after weaning. Archaea richness is increased at least until month 6 and is consistent with previous observations according to Dill-McFarland et al. (2017, 2019), who documented proper archaea establishment between weaning and 1 year of age. While archaea community composition was more similar between calves during the pre-weaning period, the variation increased over time. In contrast, some previous studies have reported individual variation to decrease with age (Dill-McFarland et al., 2017, 2019). However, due to the restricted number of reads per sample, particularly in the pre-weaning phase, it is essential to exercise caution when interpreting the results.

*Methanobrevibacter* dominated the pre-weaning community and persisted after weaning, gradually accompanied by other archaea. *Methanocorpusculum* appeared only after weaning, which suggests that they are associated with solid diet. These findings align with previous studies (Dill-McFarland et al., 2017, 2019; Li et al., 2022). *Methanocorpusculum* is more commonly found in feces than in the rumen (Tapio et al., 2016; Holman and Gzyl, 2019). They exhibit flexibility in energy precursors, utilizing CO_2_, H_2_, and formate, and in some species, secondary alcohols were used as electron donors (Chong and Boone, 2015). These archaea also possess gene-encoding bile salt hydrolases (Lin et al., 2023), potentially aiding in their adaptation to the hindgut. *Methanobrevibacter gottschalkii* was generally more abundant in feces compared with *Mbb. ruminantium*. Unlike *Mbb. ruminantium, Mbb. gottschalkii* is known to be bile tolerant (Miller, 2002). Previous studies have suggested a negative interaction between *Mbb. gottchalkii* and *Mbb. rumimantium* clades in the rumen environment, possibly due to different expression levels of methyl-coenzyme reductase I (MCRI) and methyl-coenzyme reductase II (MCRII) isozymes (Kittelmann et al., 2013). These enzymes, crucial for the final step in methane production, are expressed differently in response to varying H_2_ and CO_2_ concentrations; MCRI is expressed in low gas concentration, and MCRII is expressed in high gas concentration (Bonacker et al., 1992). Some *Mbb. ruminantium* strains (i.e., M1) possess only MCRI operon, while many other *Methanobrevibacter* species have both MCRI and MCRII operons (Leahy et al., 2010; Pitta et al., 2022).

In this study, *Methanobrevibacter* sp. was a part of the colostrum core community, which seeded in feces from 5th week onwards. Compared with milk bacteria, the milk archaea have been less studied. However, recent evidence suggests that there is a rich community of archaea in milk (Hoque et al., 2019; Shinozuka et al., 2021), and archaea have also been found in the uterus (Yeoman et al., 2018) and GI tract of calves right after birth (Guzman et al., 2015). Our results suggest that colostrum could serve as a seeding source for some archaea, but the majority were seeded from young animal rumen early in life and remained in the large intestine thereafter. However, further studies are needed to elucidate the seeding sources and succession of archaea in the hindgut.

### Anaerobic fungi seeding sources and community development

Anaerobic fungi establishment in the hindgut of ruminants is less explored compared with the rumen. Consequently, we investigated how the community of anaerobic fungi matures in the hindgut of growing animals and whether early-life rumen modulation has an impact on fecal fungal establishment. We were unable to detect anaerobic fungi in feces before weaning. This implies that the anaerobic fungi colonization in the large intestine requires transition into solid feeds, particularly intake of silage, with fungal richness increasing until month 10. Our results agree with a previous study, indicating that fecal anaerobic fungi establish sometime after weaning but before 1 year of age (Dill-McFarland et al., 2019). In a study using culturing techniques, anaerobic fungi were detected in feces of 4-week-old calf, with all studied animals acquiring fecal fungi by the age of 15 weeks (Theodorou et al., 1993). In contrast to our results and the earlier observations, a recent study suggested the presence of fecal anaerobic fungi as early as day 1 after birth, with quantities increasing until month 4 (Jones et al., 2023).

The anaerobic fungi colonization in feces is age related. The two pre-weaning samples from the T-group were mainly comprised of *Piromyces 2*, uncultured *KF1*, and uncultured *SK3* clades. Certain *Piromyces* strains have demonstrated robust growth on various substrates, including lactose and starch (Teunissen et al., 1992). It is possible that the pre-weaning diet, consisting of milk replacer along with concentrate and silage, provided favorable substrates for the growth of these fungi. Month 4 appeared as a transition time-point with strong predominance of *Caecomyces*, while starting from month 6, dominance was more evenly distributed among five fungal genera, with *BlackRhino* replacing *Caecomyces* as the most abundant group. Although originally isolated from Black rhinoceros, the *BlackRhino* clade was shown to be identical to the cultured *Piromyces* sp. SFH682 (Fliegerova et al., 2021). Considering the similarity of *BlackRhino* to *Piromyces*, our results agree with Jones et al. (2023) who showed that the post-weaning fungal communities had higher abundance of *Caecomyces*, *Piromyces*, and *Cyllamyces* in comparison to the pre-weaning communities. Our results also indicated that different *Piromyces* species might be accustomed to the hindgut environment at different age stages in growing animals. From month 8 onwards, while *BlackRhino* (*Piromyces*) continued to have high relative abundance, other *Piromyces* species began to decrease, disappearing almost entirely from the feces by month 12. This time-point coincided with a shift in the maintenance diet to a lower-energy diet with higher roughage content (Huuki et al., 2022b). Because easily fermented carbohydrates are, to a large extent, consumed in the rumen, and in some cases *Piromyces* have been observed to degrade straw less efficiently than *Neocallimastix* and *Caecomyces* (Gordon and Phillips, 1989), it is possible that some *Piromyces* species were not able to compete on the remaining available substrates.

Previously, we demonstrated that a proper establishment of anaerobic fungi in the rumen of dairy calves took place only after weaning and reached the mature-stable state at approximately 10 months age (Huuki et al., 2022a,b). When young animals’ own rumen was evaluated as a seeding source, we demonstrated that from month 4 onwards, both rumen and feces shared the same fungal taxonomical groups; only different fungal species showed predominance in feces as compared with the rumen. For example, *Neocallimastix* 1 was the most abundant clade after month 4 in rumen samples (Huuki et al., 2022b), while *BlackRhino* (*Piromyces*) group and *Caecomyces* 1 group were among the most abundant taxa in fecal samples. Recently, Meili et al. (2023) have shown evidence of the phylogenetic co-evolution of anaerobic fungal taxa, where *Piromyces* show preference for hindgut fermenters, while more recently evolved *Neocallimastix* may have co-evolved with the rumen. *Caecomyces* are found in both foregut and hindgut fermenters (Meili et al., 2023) and have been found to be more abundant in fecal samples of cows in comparison to the rumen (Tapio et al., 2016).

The anaerobic fungi transfer in the developing rumen might be also facilitated by the shared environment, and to a large extent, the fungal community is shared by weaned young and adult animals within the barn, while the inter-animal differences in abundances of different taxa are caused by host effects (Huuki et al., 2022a, 2022b). Indeed, anaerobic fungi are found in the mouth (Lowe et al., 1987; Tapio et al., 2016), and due to resistant structures, they can be viable months after defecation (McGranaghan et al., 1999) and can therefore be easily transmitted through contact and cross-contamination of bedding and feed with feces. In this study, when the heifers were moved into a different facility after month 4, we observed an increase in the rumen and fecal fungal diversity. The changes in the community composition were likely affected by dietary and host-related factors, such as functional development and the growth of the rumen and intestine. Nevertheless, the exposure to the new fungal taxa in the new barn environment may have contributed to the increase in taxonomical diversity. Therefore, we hypothesize that the rumen is the direct seeding source for fungi in feces, and the colonization of both the rumen and feces takes place around the same time, and that the fungi are being continuously seeded from the environment to the rumen, and when entering the hindgut, the hindgut conditions, diet, and interaction with other microbes finally determine the community structure.

### Effects of inoculum treatment on community composition and gut health

During the pre-weaning period, some of the calves in both groups experienced diarrhea. The diarrhea can be caused by bovine rotavirus, coronavirus, norovirus, and bacteria such as *Salmonella*, *Escherichia coli*, *Clostridium perfringens*, or protozoa *Cryptosporidium parvum*, which cause dysbiosis of microbial community (Cho and Yoon, 2014). In the present study, diarrhea reduced the fecal bacterial diversity in calves, which is in line with previous observations (Gomez et al., 2022). Our results showed that the reduction of diversity in diarrhea cases was associated with the lower relative abundance of beneficial genera, e.g., *Bifidobacterium* and *Faecalibacterium* and with simultaneously increased potential pathogenic genera such as *Escherichia-Shigella, Streptococcus*, *Clostridium sensu stricto 1*, and *Lactobacillus*. *Faecalibacterium* and *Bifidobacterium* are butyrate-producing bacteria that, in humans, promote a balanced microbial community, reinforce the gut barrier, produce anti-inflammatory substances, competitively exclude pathogens, and modulate immune responses (O’Callaghan and Van Sinderen, 2016; Martín et al., 2023). The decrease in butyrate-producing taxa (*Faecalibacterium* and *Bifidobacterium*) and the increase in facultative anaerobic lactic acid-producing taxa (e.g., *Escherichia-Shigella*, *Streptococcus*) in diarrhea cases could be related to increased oxygen, leaking into the hindgut lumen, which inhibits the growth of oxygen-sensitive beneficial obligate anaerobes, and decreased pH and increased lactate production in the gut, which promotes the growth of facultative anaerobes such as *Escherichia-Shigella* and *Streptococcus* (Gomez et al., 2022). However, our results showed that the beneficial taxa associated with healthy samples disappeared from the feces of all calves before weaning, and the potential pathogenic taxa by month 6, suggesting that their negative impact and role in the gut health is restricted to early life period only.

We hypothesized that early-life rumen microbiota modulation could also modulate hindgut microbiota and be beneficial to gut health, as the donor inoculum promoted microbiota development in the rumen (Huuki et al., 2022a,b). However, despite effects to the rumen, the treatment neither reduced diarrhea cases nor showed differences between the groups in fecal bacterial community composition before and after diarrhea. Treatment did not have a marked impact on early-life fecal microbiota colonization either and did not serve as a major seeding source of fecal microbiota. In the present study, the inoculum originated from adult cows’ rumen which is well adapted to plant fiber fermentation, while some special characteristics (such as gastric acid and bile tolerance and/or alternate substrates for energy source) are needed for the microbes to pass the abomasum and survive in the lower intestine. Previous studies have noted that inoculation with rumen liquid (Bu et al., 2020) or fecal microbiome transplantation (FMT) (Kim et al., 2021; Rosa et al., 2021) may reduce diarrhea in young ruminants, and FMT has an effect on the fecal microbiota composition. The microbial communities in fecal transplant have already adapted to the hindgut environment, which may have contributed to the slightly better success in treating diarrhea with FMT treatment. Therefore, the target environment and the age of the animal should be taken into account when designing microbial inoculations to improve gut health, gut development, or production performance.

## Conclusion

This study highlights the plasticity of the GI tract microbial colonization, where crosstalk between the colostrum, rumen, and hindgut allows the GI tract to adjust in time and space to changing dietary and developmental needs. We showed that the colostrum may be a source for some of the hindgut bacteria and archaea, but their role in the gut mostly is restricted to the pre-weaning period. In comparison to the rumen, the fecal bacterial community matures earlier, potentially due to a more stable environment. Although present in the feces of pre-weaning calves, archaea increased in quantity and diversity after weaning when animals shift to utilizing the solid feeds only. Anaerobic fungi in feces occurred after weaning, and the diversity increased until reaching adulthood. The fecal anaerobic fungi are seeded through the rumen, and the communities of the rumen and large intestine mature at the same time. Despite early-life rumen microbiota modulation having a positive effect on rumen microbiota development, no effect was observed either on the development of fecal microbiota or on the pre-weaning gut health. The future applications of gut microbiota modulation should be designed taking into account the intended trait, gut region, and the age of the animal, e.g., rumen and hindgut communities have adapted to different environments with different niches.

## Data availability statement

The datasets presented in this study can be found in online repositories. The names of the repository/repositories and accession number(s) can be found below: https://www.ncbi.nlm.nih.gov/, BioProject PRJNA713003.

## Ethics statement

The animal study was approved by Project Authorisation Board (Regional Administrative Agency for Southern Finland, Hämeenlinna, Finland; ESAVI/17310/2021). The study was approved by the National Ethics Committee (ESAVI/5687/04.10.07/2017, Hämeenlinna, Finland) The study was conducted in accordance with the local legislation and institutional requirements.

## Author contributions

HH: Data curation, Formal analysis, Investigation, Methodology, Visualization, Writing – original draft. JV: Conceptualization, Writing – review & editing. AV: Supervision, Writing – review & editing. IT: Conceptualization, Funding acquisition, Investigation, Project administration, Resources, Supervision, Writing – original draft.

## Supplementary material

SUPPLEMENTARY FIGURE S1 The Shannon diversity **(A)** and the Simpsons diversity index **(B)** of fecal becteria communities in rumen liquid inoculated (T) and control animals **(C)** from week 1 to month 12 of age.

Supplementary FIGURE S2 The relative abundances of bacterial phyla in fecal samples of rumen liquid inoculated (T) and control heifers **(C)** from week 1 to month 12 of age.

Supplementary FIGURE S3 The Shannon diversity **(A)** and the Simpsons diversity index **(B)** of fecal archaea communities in rumen liquid inoculated (T) and control animals **(C)** from week 1 to month 12 of age. Statistical analysis was performed for month 4-12 samples only, due to low number of reads and missing observations in pre-weaning samples.

Supplementary FIGURE S4 The Shannon diversity **(A)** and the Simpsons diversity index **(B)** of fecal anaerobic fungi in rumen liquid inoculated (T) and control animals **(C)** from month 4 to month 12 of age.

Supplementary FIGURE S5 The average relative abundance of differently abundant ASVs between healthy samples (green) and samples with diarrhea symptoms (red) in samples from weeks 2 to 4.

Supplementary FIGURE S6 The average contribution of own mothers’ colostrum, shared colostrum mix, donor inoculum and calves’ own rumen community at 2, 4, 6, and 8 weeks, and at 4 months age as a seeding source to fecal bacteria community in calves at the age from 1 week to 4 months.

Supplementary FIGURE S7 The average contribution of donor inoculum and heifers’ own rumen community between ages from 4 to 9 months as a seeding source to the fecal fungi community of heifers the age from 1 week to 4 months.
