# Supplementary figures and images for: Fecal microbiota colonization dynamics in dairy heifers associated with early-life rumen microbiota modulation and gut health
Source: Front Microbiol. 2024 Mar 5;15:1353874. doi: 10.3389/fmicb.2024.1353874 (PMC10949896; doi:10.3389/fmicb.2024.1353874)

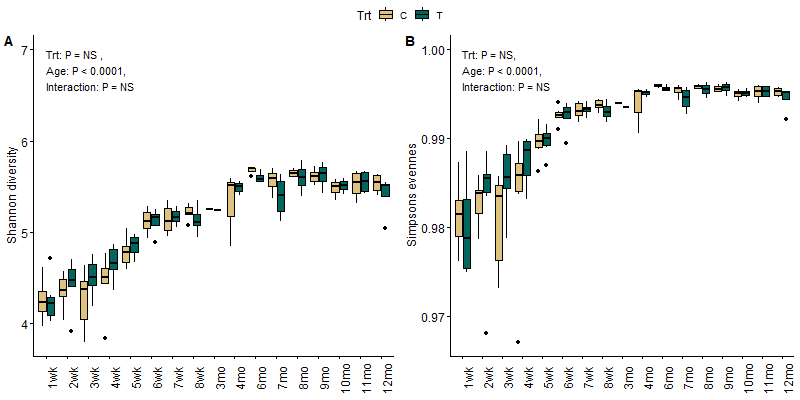

Supplement: SUPPLEMENTARY FIGURE S1 — The Shannon diversity (A) and the Simpsons diversity index (B) of fecal becteria communities in rumen liquid inoculated (T) and control animals (C) from week 1 to month 12 of age. [file Image_1.TIFF]

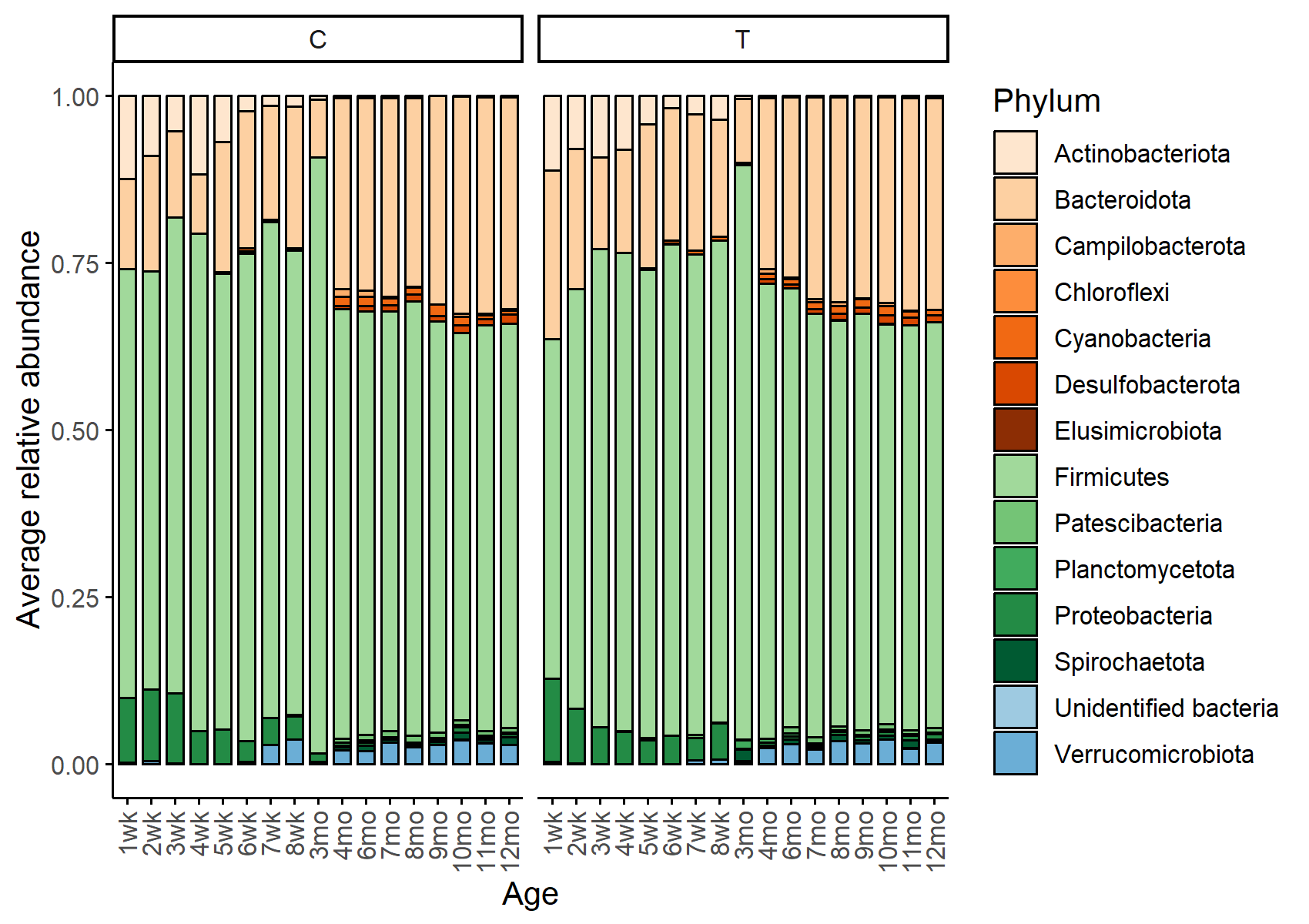

Supplement: Supplementary FIGURE S2 — The relative abundances of bacterial phyla in fecal samples of rumen liquid inoculated (T) and control heifers (C) from week 1 to month 12 of age. [file Image_2.TIFF]

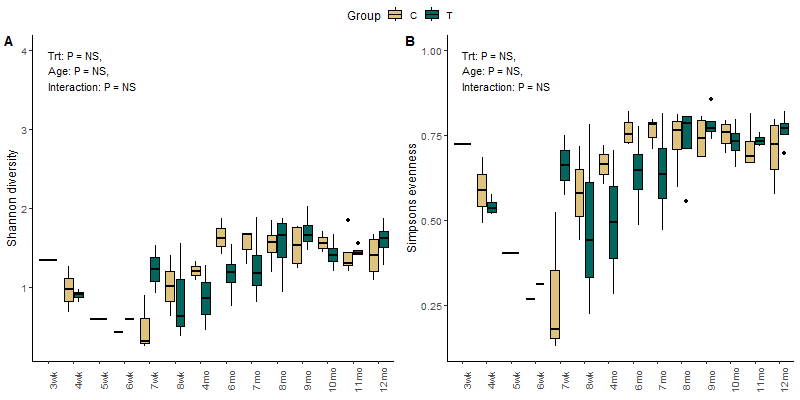

Supplement: Supplementary FIGURE S3 — The Shannon diversity (A) and the Simpsons diversity index (B) of fecal archaea communities in rumen liquid inoculated (T) and control animals (C) from week 1 to month 12 of age. Statistical analysis was performed for month 4-12 samples only, due to low number of reads and missing observations in pre-weaning samples. [file Image_3.TIFF]

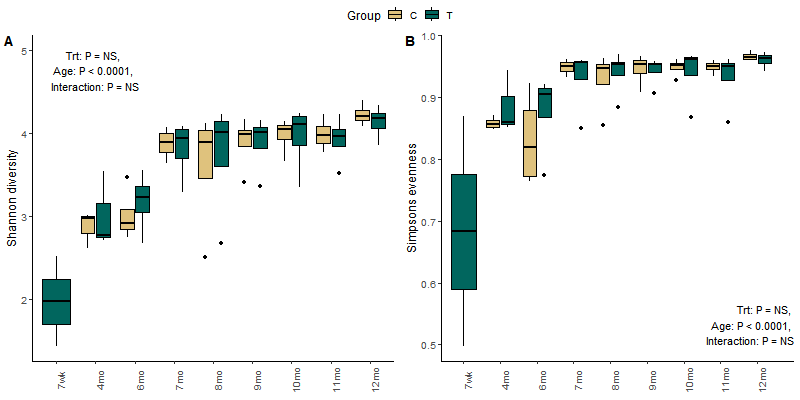

Supplement: Supplementary FIGURE S4 — The Shannon diversity (A) and the Simpsons diversity index (B) of fecal anaerobic fungi in rumen liquid inoculated (T) and control animals (C) from month 4 to month 12 of age. [file Image_4.TIFF]

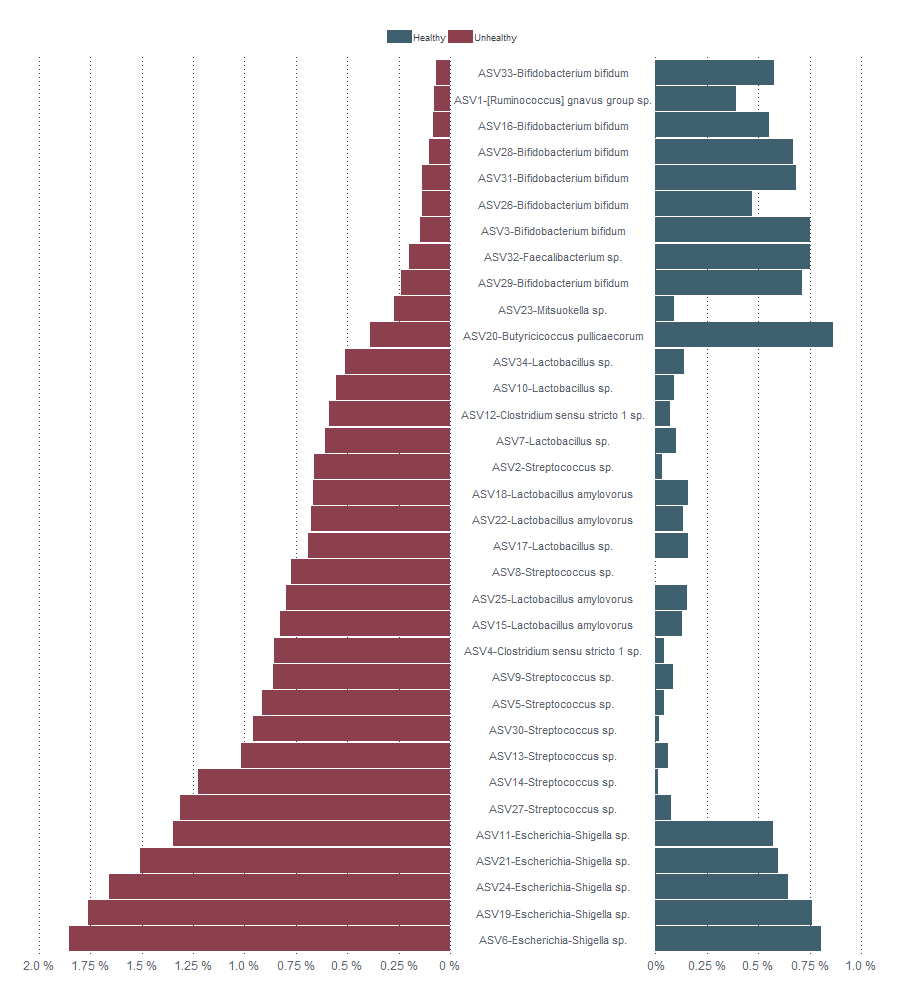

Supplement: Supplementary FIGURE S5 — The average relative abundance of differently abundant ASVs between healthy samples (green) and samples with diarrhea symptoms (red) in samples from weeks 2 to 4. [file Image_5.TIFF]

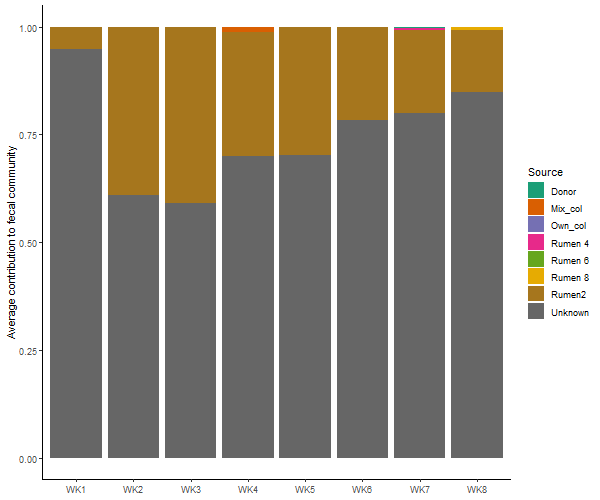

Supplement: Supplementary FIGURE S6 — The average contribution of own mothers’ colostrum, shared colostrum mix, donor inoculum and calves’ own rumen community at 2, 4, 6, and 8 weeks, and at 4 months age as a seeding source to fecal bacteria community in calves at the age from 1 week to 4 months. [file Image_6.TIFF]

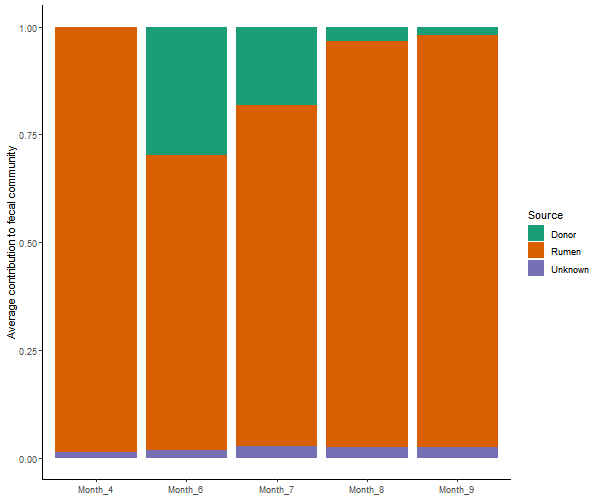

Supplement: Supplementary FIGURE S7 — The average contribution of donor inoculum and heifers’ own rumen community between ages from 4 to 9 months as a seeding source to the fecal fungi community of heifers the age from 1 week to 4 months. [file Image_7.TIFF]
